# Air Pollution and Health: A Science-Policy Initiative of National Academies

**DOI:** 10.5334/aogh.2670

**Published:** 2019-12-16

**Authors:** Luiz Davidovich, Paulo Saldiva

**Affiliations:** 1 Brazilian Academy of Sciences, BR; 2 Institute of Advanced Studies of the University of São Paulo, BR

Air pollution is a major cause of disease. It is estimated that, globally, at least 5 million people die prematurely due to bad air quality. Although pollution is a global problem, the health burden is not evenly distributed across countries. Underdevelopment is often linked with bad air. Emissions produced by low-technology devices used for cooking and heating deteriorate household air quality. Outdoors, air pollutants result from excessive use of fossil fuels by inefficient motor engines or thermoelectric power plants, making urban streets and their vicinity an unhealthy space for urban dwellers. Traffic congestion, a consequence of urban transportation systems, poses an additional risk to urban commuters, because they are unavoidably exposed for longer periods of time to the smoky exhaust pipes in traffic jams.

The picture that describes the health effects of air pollution was painted with scientific tinctures by a global team of scientists working in many institutions located in all continents. The image created by this collective work was not pleasant, but it was realistic. Air pollution is unequivocally associated with significant morbidity and mortality due to different conditions, including cardio-respiratory diseases, lung cancer, and low birth weight. Scientific evidence clearly indicates the necessity of immediate actions to reduce air pollution. Public policies are needed, because air pollution is conspicuous and cannot be avoided by individual attitudes or procedures. Thus, the reduction of health burden caused by air pollution depends on public policies. Scientists representing national academies of four continents have formed a coalition, this time not to produce new data or evidences, but to discuss solutions and directions for those in charge of leading the destiny of our planet. The statement points towards directives aimed to increase the use of cleaner energy sources and engines, new strategies to favour low emissions in individual and public transportation, and intelligent urban planning and design. Scientists have also demonstrated that the proposed solutions are not only already available, but also feasible and cost-effective. The reduction of health costs and the loss of years of life because of illness or premature death largely outweighs the monetary resources needed for their implementation. Science has demonstrated that reducing local air pollution leads to the reduction of greenhouse gas emissions, thus contributing to the fight against global warming and climate change.

Science has described and quantified the health effects of air pollution. Scientists have developed the technological solutions to reduce air pollution and demonstrated the economic viability of cleaning the air we breathe. Now the five academies established the pathway for science-based policies necessary to achieve a better air quality. Policies for reducing air pollution extrapolate the limits of health science by including the subjects of social equity, human rights, and global sustainability. Scientists have done their job. It is time for politicians to get on stage.

